# Sex-differences in psychological readiness for return-to-sport following anterior cruciate ligament reconstruction

**DOI:** 10.1371/journal.pone.0307720

**Published:** 2024-09-18

**Authors:** Turk Robby, Nadim Hussein, Arakua Welbeck, Mallory Faherty, Carolyn Killelea, Lee Diehl, Jocelyn Wittstein, Jonathan Riboh, Alison Toth, Ned Amendola, Timothy C. Sell

**Affiliations:** 1 Department of Orthopaedic Surgery, Atrium Health Carolinas Medical Center, Charlotte, NC, United States of America; 2 Department of Orthopaedic Surgery, Duke University School of Medicine, Durham, NC, United States of America; 3 OhioHealth Research Institute, OhioHealth, Columbus, Ohio, United States of America; 4 OrthoCarolina Sports Medicine, Charlotte, NC, United States of America; 5 Atrium Health Musculoskeletal Institute, Charlotte, NC, United States of America; Ningbo University, CHINA

## Abstract

Females are at greatest risk for reinjury after return to sport (RTS) following anterior cruciate ligament (ACL) reconstruction (ACLR). The reasons for these sex differences, however, remain unclear. Psychological factors such as kinesiophobia have been identified as a potential predictor for reinjury following RTS. Studies investigating kinesiophobia have identified sex differences, yet whether this holds in the ACLR population remains unknown. The purpose of this study was to examine whether there are sex differences in kinesiophobia and other psychological factors, such as readiness to RTS and self-reported pain in the ACLR population. A total of 20 participants, eleven males (23.0 ± 8.4 years, 178.9 ± 7.6 cm, 76.8 ± 10.4 kg) and 9 females (19.6 ± 5.3 years, 165.1 ± 4.0 cm, 73.2 ± 25.0 kg) voluntarily participated in this study. The Tampa Scale for Kinesiophobia (TSK-11), Anterior Cruciate Ligament Return to Sport After Injury (ACL-RSI) scale, and self-reported pain using a visual analog scale (VAS) were administered after clearance for RTS (10.5 ± 2.3 months post-ACLR). Statistical significance was set *a priori* at p<0.05. A significant difference between sexes was observed for the ACL-RSI with males reporting a significantly higher score (92.82±16.16) compared to females (77.0±15.54; p = 0.040). There were no significant differences between sexes for VAS for pain (males = 4.55 ± 6.50; females = 1.22 ± 3.31; p = 0.228) and TSK-11 (males = 18.73 ± 3.17; females = 19.67 ± 4.61; p = 0.596). The results of this study demonstrated males had significantly higher ACL-RSI scores than females, suggesting males may have higher psychological readiness following clearance for RTS. This study did not demonstrate significant differences between sexes for kinesiophobia or pain level. Caution in interpretation of results is warranted due to the small sample size, highlighting the need for further research in this area.

## Introduction

The anterior cruciate ligament (ACL) is one of the most common sports related injuries in orthopaedics with over 200,000 cases reported annually in the United States [1]. The majority of ACL injuries are noncontact injuries that occur due to anatomic, biomechanical, neuromuscular, hormonal, and other risk factors which continue to be identified in the literature [2, 3]. The gold standard treatment for an ACL rupture in an athlete is early anatomic ACL reconstruction (ACLR) with an autologous graft typically obtained from the patellar, hamstring, or quadriceps tendons [4]. Following ACL reconstruction surgery (ACLR), less than 50% of competitive athletes return to their previous level of activity [5].

Over one-third of athletes will suffer a second ACL injury to the ipsilateral ACL graft or contralateral ACL within the first 24 months after ACLR [1, 5–7]. The documented differences in second ACL injury rates between males and females are particularly concerning [7–9]. Although the early studies are conflicting, evidence indicates that females may be more susceptible to reinjury than males [7, 10, 11]. These sex differences in re-injury rates would suggest that there are sex-specific risk factors for second ACL injury. Many different risk factors for second ACL injury have been explored, including sex-specific risk factors [12–15]. Psychological factors such as pain perception, kinesiophobia, and psychological readiness to return to sport (RTS) are increasingly being explored due to their importance to success or failure following ACLR [11, 16–18].

One psychological consideration that has been implicated as a risk factor is kinesiophobia, which is the fear of pain due to movement [19]. Patients with higher levels of kinesiophobia are less active during rehabilitation, perform worse during performance testing, including single-hop test and quadriceps strength, and ultimately have a higher risk of second ACL injury [17]. Other psychological factors may also play a role in a second ACL injury, including psychological readiness to return to sport and pain. Anterior cruciate ligament injury can have a profound psychological effect on an athlete, and a negative psychological effect before returning to sport can lead to reduced sports participation or potential reinjury. For example, males have been demonstrated to have a higher psychological readiness to return to sport (RTS) than females, who also have a higher prevalence of not returning to their sport following ACLR [10, 20, 21]. Furthermore, there are documented sex differences in pain [22–24]. These differences have yet to be fully explored in ACLR populations.

The primary purpose of this study was to determine if there are sex differences in kinesiophobia, readiness to return to sport, and self-reported pain in individuals who have undergone ACLR after clearance for RTS. We hypothesized that there would be sex differences, with males demonstrating lower kinesiophobia, greater psychological readiness to return to sport, and lower pain assessments than females. As the causes underlying the sex differences in ACLR reinjury rates are not completely understood, the results of this study may provide a better understanding of the psychological factors that impact reinjury. The results of this study, if sex differences are observed, may inform targeted, sex-based interventions focused on returning individuals to sport with less risk of second ACL injury.

## Materials and methods

### Participants

All subjects voluntarily signed informed consent as approved by Duke University IRB. Subjects were recruited from a regional group of fellowship trained orthopedic surgeons with similar guidelines and protocols for ACLR rehabilitation. All subjects underwent ACLR between August 2017 and August 2018. The first patient was enrolled on June 13^th^, 2018. The final patient was enrolled on August 12^th^, 2019. Patients eligible for the study were 12 years or older with unilateral ACL tear and consequent reconstruction. Those with concomitant meniscal or multi-ligament involvement were not eligible. A total of 20 participants who fit these parameters voluntarily participated in the study (Table 1). These participants were part of an ongoing study examining risk factors for second ACL injury and successful return to sport.

**Table 1:** Demographics of study participants.

		Combined			Females				Males					
	n	Mean	Median	SD	n	Mean	Median	SD	n	Mean	Median	SD	p-value	Effect Size
**Age (years)**	**20**	**21.5**	**18.3**	**7.2**	**9**	**19.6**	**18.0**	**5.3**	**11**	**23.0**	**19.6**	**8.4**	**0.342**	**na**
**Height (centimeters)**	**20**	**172.7**	**170.4**	**9.3**	**9**	**165.1**	**167.0**	**4.0**	**11**	**178.9**	**176.2**	**7.6**	**p≤0.001**	**2.190**
**Mass (kilograms)**	**20**	**75.2**	**74.4**	**18.0**	**9**	**73.2**	**64.5**	**25.0**	**11**	**76.8**	**75.1**	**10.4**	**0.184**	**na**
**Time until Return to Sport (months)**	**20**	**9.5**	**9.0**	**2.6**	**9**	**8.7**	**9.0**	**2.7**	**11**	**10.1**	**8.9**	**2.5**	**0.2513**	**na**

Abbreviations: na = not applicable, SD = standard deviation, n = sample size

### Data collection

Patient Reported Outcomes (PRO) were collected via survey administered in-person at the time of clearance for RTS (10.21 ± 2.23 months post-ACLR). These included several widely used questionnaires that have been verified in ACLR populations including the Tampa Scale for Kinesiophobia (TSK-11) [25, 26], the Anterior Cruciate Ligament Return to Sport After Injury (ACL-RSI) scale, and visual analog scale (VAS) for pain [21]. The TSK-11 is an 11-item questionnaire and uses a 4-point Likert scale with scoring alternatives ranging from “strongly disagree” to “strongly agree. Four of the items are inversely scored and the scores range from 17–68 with higher scores representing greater fear of reinjury with physical movement and lower scores representing less fear [25, 26]. The ACL-RSI scale is a 12-item questionnaire using a 10-point visual analog scale used to assess psychological readiness to return to sport with a focus on psychological responses associated with emotions, confidence in performance and risk appraisal. The scores may range from 0–100 with a lower score indicating more negative psychological responses while higher scores represent less negative psychological responses [20, 27]. The VAS scale for pain uses a score from 0 to 10 with a higher score representing more pain and a lower score representing less pain [28, 29].

### Statistical analysis

Statistical analysis began by calculating summary statistics for all variables, including mean, median, and standard deviation. Summary statistics were calculated for the entire cohort, males and females. All data were assessed for normality utilizing Shapiro-Wilk before statistical analysis. Comparisons between males and females were performed using parametric or non-parametric tests based on the results of the Shapiro-Wilk analysis. Independent samples t-tests were performed when the data were determined to be normally distributed. Age, height, time to RTS, ACL-RSI, and TSK-11 were normally distributed, so independent samples t-tests were performed to compare groups. Wilcoxon rank-sum tests were utilized to make group comparisons for age, weight, and VAS since each variable was not normally distributed. Statistical significance was set *a priori* at p<0.05. Effect size was calculated for any variable that resulted in significant differences between groups (Cohen’s d). All statistical calculations and statistical analyses were performed with STATA (version 17 (StataCorp LLC (College Station, TX))).

## Results

There were no differences in age, weight, or time until RTS between the males and females. There was a significant difference between groups for height, with males being taller than females. The effect size for this difference was 2.19, indicating a large effect size as the two groups differ by greater than two standard deviations. The descriptive statistics and results of the comparisons between groups for ACL-RSI, Pain VAS, and TSK-11 are reported in Table 2. A significant difference was found in ACL-RSI values between male and female participants (males = 92.82 ± 16.16 and females = 77.0 ± 15.54), with males having significantly higher ACL-RSI than females. The effect size for this difference was 0.996, indicating a large effect size [30] as the two groups differ by approximately one standard deviation. This represents the standardized difference between the means of males and females. This would represent approximately 17 points as measured by the ACL-RSI. No significant differences were found between males and females in terms of the Pain VAS and TSK-11 outcomes.

**Table 2:** Patient reported outcomes.

		Combined				Females				Males				
	n	Mean	Median	SD	n	Mean	Median	SD	n	Mean	Median	SD	p-value	Effect Size
**Pain Visual Analog Scale**	**20**	**3.05**	**0.00**	**5.45**	**9**	**1.22**	**0.00**	**3.31**	**11**	**4.55**	**0.00**	**6.50**	**0.228**	**na**
**Tampa Scale for Kinesiophobia 11**	**20**	**19.15**	**19.00**	**3.80**	**9**	**19.67**	**19.00**	**4.61**	**11**	**18.73**	**19.00**	**3.17**	**0.596**	**na**
**Anterior Cruciate Ligament—Return to Sport After Injury Scale**	**20**	**85.70**	**85.50**	**17.44**	**9**	**77.00**	**70.00**	**15.54**	**11**	**92.82**	**94.00**	**16.16**	**0.040**	**0.996**

Abbreviations: na = not applicable, SD = Standard Deviation, n = sample size

## Discussion

The primary purpose of this study was to determine if there are sex differences in kinesiophobia, readiness to return to sport, and self-reported pain in individuals who have undergone ACLR after clearance for RTS. We hypothesized that there would be sex differences, with males demonstrating lower kinesiophobia, greater psychological readiness to return to sport, and lower pain assessments than females. Consistent with the author’s hypothesis, there was a significant difference in readiness to return to sport, with males having higher psychological readiness to return to sport (ACL-RSI) than females following ACLR. However, male and female patients had similar reported outcomes of kinesiophobia and pain perception which did not support the original hypothesis. These findings provide critical insight into the psychological factors that may impact functional outcomes, return to sport, and rates of second injury following ACLR.

The major findings from this study parallel those of Webster et al. who assessed psychological readiness to return to sport in the ACLR population using the ACL-RSI and had similar results, concluding females had lower ACL-RSI scores than males [21]. In this study, they also found females to have lower rates of return to sport, indicating that psychological readiness is a significant contributing factor to returning to previous level of sport, a finding that has been supported by a number of studies to date [11, 16]. Paterno et al. also found psychological readiness to effect rates of reinjury after ACLR in a study which demonstrated that patients with greater self-reported fear of ACL reinjury had lower strength, lower activity, and higher likelihood of ACL reinjury in the 24 months after ACLR in a study [17]. Another study by Baez et al. found that patients who had a Tampa scale of Kinesiophobia-11 (TSK-11) score of 19 were 13 times more likely to suffer a second ACL tear within the 24 months following initial ACLR and suggested psychological factors to be more closely associated with RTS than functional outcomes [31]. In this context, the results of the present study provide data which supports the idea that female patients may be at increased risk for second injury as well as not returning to their previous level of sport following ACLR.

The results of this study are not without their counterparts in the respective literature. While this study found no difference in kinesiophobia and pain related PRO’s (TSK-11 and VAS, respectfully) between the populations in question, Van Velzen et al. identified a sex-difference in TSK-11 while Bartley and Fillingim found a similar difference in self-reported VAS Pain [22, 24]. These studies examined these outcomes outside of the ACLR population, however, still provide useful additional context given the lack of evidence currently available in the ACLR population. Given the number of complex variables and implications associated with these studies, additional work and data assessing larger patient populations are needed to achieve external validity of these findings for the ACLR population. This could include studies examining individuals who have undergone ACLR reconstruction prospectively and longitudinally with a focus on kinesiophobia and surveys of pain.

The reason for these sex-based differences in RTS after ACLR is unclear and likely multifaceted. As previously mentioned, there is a well-studied difference in pain perception between men and women in population studies which may also present itself following ACLR. It may also be related to coping and other complex mechanisms which currently have limited evidence in the literature. Hartigan et al. defined coping as an important component of ACLR recovery, however they also illustrated that kinesiophobia was a significant aspect of postoperative ACLR rehabilitation regardless of whether the patient was inclined to cope with potential changes in knee function after ACLR [32]. The underlying cause of these sex-differences demonstrated in the findings of this study remains a critical area of exploration for future studies.

The present results have various implications for both preoperative and postoperative interventions which could potentially improve patient outcomes following ACLR. Although the results of the present study require additional validation and further investigation, it raises the question of whether or not sex-specific treatment, such as allowing for a shorter time from injury to surgery for males, and a longer time for females, may improve both psychological and functional outcomes following ACLR. Postoperative interventions such as psychological coaching and consistent psychological readiness assessments in females, or potentially both sexes, may also be valuable interventions that warrant future study. It is critical to note that these implications fundamentally reflect the importance of individualized RTS decisions based on surpassing objective thresholds of a given RTS criteria rather than solely based on time since surgery- an approach which has also been strongly supported in the literature.

The greatest limitation of the current work was the sample size. The total of 20 patients in the study population necessitates evaluating the results within the context of additional works. A sample size estimate was not performed for the current analysis as these subjects were part of a prospective, longitudinal study and were the first participants enrolled as part of this larger study. The objective was to gain some knowledge sooner relative to potential sex differences in kinesiophobia and the other surveys including in the current study. However, this is a well-organized study with clinically significant outcomes that provide relevant information to be utilized within larger analyses or by itself. An additional limitation is that participants were gathered from multiple different institutions and therefore cannot be confirmed to have had the same intraoperative techniques or postoperative care. However, given the similarities of ACLR outcomes and postoperative ACLR protocols, it is likely that these protocols did not vary in a way that would significantly impact the outcomes of interest. Lastly, patients over 12 years old were included, meaning this study did not control for physiological bone age and patients with variable stages of growth plate closure, which may indicate different graft selections and ACLR techniques. Nevertheless, other studies investigating this topic have also not controlled for age and have still provided clinical utility as demonstrated by the previously mentioned study by Ryan et al. and others [33]. Finally, recovery, rehabilitation, return to sport, and pain processing following ACLR is complicated and is affected by many different variables. The current paper only includes a few of these variables and should be interpreted carefully.

This cohort study illustrates and examines sex-differences in a number of clinically important psychological factors following ACLR. The results of this study may help to shape RTS decision making and rehabilitation protocol by supporting sex-specific rehabilitation protocols following ACLR. Given the literatures support for increased rates of reinjury and decreased RTS for female patients, addressing the differences in physical and psychological parameters such as is indicated here may help to eliminate this disparity. This could include incorporating increased amounts of psychological coaching and/or evaluation, higher proportion of sport-specific rehabilitation training, and/or addition of psychological testing as a routine part of RTS protocol for women following ACLR. If psychological factors associated with RTS are noted to improve in a linear fashion with time, it could also suggest a later RTS time for female patients would help optimize outcomes. By adding to the growing levels of data available on this topic, this study further supports the clinical utility of assessing and providing interventions for psychological factors in ACLR patients with the aim to improve functional outcomes, increase rate of RTS, and decrease repeat injury rates following ACLR.

## Supporting information

S1 File (PDF)
