# Supplementary material for: Sex-differences in psychological readiness for return-to-sport following anterior cruciate ligament reconstruction
Source: PLoS One. 2024 Sep 18;19(9):e0307720. doi: 10.1371/journal.pone.0307720 (PMC11410221; doi:10.1371/journal.pone.0307720)
Supplement: S1 File — (PDF) [file pone.0307720.s001.pdf]

Subject Number	Gender	Return to Sport Time in Months	Age in Years	Height in Centimeters	Weight in Kilograms	Pain Scale Score	Anterior Cruciate Ligament Return to Sport after Injury Total Score	Tampa Scale of Kinesiophobia Total Score
1	Male	8.482	23.8575	189.2	83.8	10	97	22
2	Male	8.877	16.1945	176.2	75.7	5	94	23
3	Male	7.463	14.9726	176.7	81	10	107	22
4	Male	12.000	17.9836	172.1	74.4	0	100	16
5	Male	8.942	19.611	188.9	74.1	0	118	16
6	Male	9.501	20.6493	168	75.1	20	72	15
7	Male	11.967	36.863	187.3	87	5	62	21
8	Male	8.712	16.5781	173.9	68.6	0	88	21
9	Male	8.055	17.8822	173.6	55.3	0	90	16
10	Male	10.816	30.7973	175	74.3	0	108	15
11	Male	16.044	38.1205	186.6	95.9	0	85	19
12	Female	8.712	18.6411	168.4	77.9	10	78	16
13	Female	11.770	15.6521	156.8	48.9	0	68	15
14	Female	10.093	17.1151	167	62.2	0	86	23
15	Female	11.277	26.5233	167	92.7	1	62	26
16	Female	10.159	18.7562	167	66.09	0	66	27
17	Female	5.786	13.5973	160.1	64.5	0	112	15
18	Female	8.252	17.9479	168.7	60.5	0	67	17
19	Female	9.041	17.7781	163.6	55.23	0	70	19
20	Female	3.156	30.1205	167	130.5	0	84	19
